# Cybercrime: Identification and Prediction Using Machine Learning Techniques

**DOI:** 10.1155/2022/8237421

**Published:** 2022-08-27

**Authors:** K. Veena, K. Meena, Ramya Kuppusamy, Yuvaraja Teekaraman, Ravi V. Angadi, Amruth Ramesh Thelkar

**Affiliations:** ^1^Department of Computer Science and Engineering, Sathyabama Institute of Science and Technology, Chennai 600119, India; ^2^Department of Computer Science and Engineering, GITAM School of Technology, GITAM-Bangalore 561203, India; ^3^Department of Electrical and Electronics Engineering, Sri Sairam College of Engineering, Bangalore 562106, India; ^4^Department of Electronic and Electrical Engineering, The University of Sheffield, Sheffield S1 3JD, UK; ^5^Department of Electrical & Electronics Engineering, Presidency University, Bangalore City 560064, India; ^6^Faculty of Electrical & Computer Engineering, Jimma Institute of Technology, Jimma University, Jimma, Ethiopia

## Abstract

In the world of cyber age, cybercrime is spreading its root extensively. Supervised classification methods such as the support vector machine (SVM) and K-nearest neighbor (KNN) models are employed for the classification of cybercrime data. Likewise, the unsupervised mode of classification involves the techniques of K-means clustering, Gaussian mixture model, and cluster quasi-random via fuzzy C-means clustering and fuzzy clustering. Neural networks are employed for determining synthetic identity theft. The formation of clusters takes place using these clustering techniques, which fetches crime data from the overall data. Cybercrime detection employs dataset that is fetched from CBS open data StatLine. The attributes utilized are concerning the crime victims through personal characteristics with total user identity being 1000. For analyzing the performance, different training and testing data undergo variation. Eventually using the best technique, the criminal is identified and the Gaussian mixture model in the unsupervised method reveals enhanced performance using the detection method. 76.56% percentage of accuracy is achieved in detecting the criminal. The accuracy achieved in case of classification via SVM classifier is 89% in the supervised method. Performance metrics for several attributes are being computed in terms of true positive (TP), false positive (FP), true negative (TN), false negative (FN), false alarm rate (FAR), detection rate (DR), accuracy (ACC), recall, precision, specificity, sensitivity, and Fowlkes–Mallows scores. The expectation-maximization (EM) algorithm is employed for assessing the performance of the Gaussian mixture model.

## 1. Introduction

In the world of cyber age, cybercrime is spreading its root extensively. The research emphasizes to attend cybersecurity from the viewpoint of access control, particularly by detecting the cyber user and reporting criminal actions to the cybercrime investigators so that they can investigate and take legal actions against the criminals. To resolve any case concerning the cybercrime, there are no data available beforehand, and hence, there occurs a need of a machine learning model, in which data can be classified precisely through analysis, and by considering the features, the prediction of the classes can be carried out. The ultimate goal is to enhance the security performance of the network so that it can be safeguarded from attackers. With the help of cluster computing techniques and real-time dataset, the performance evaluation of several cybercriminal detection methods is analyzed. The assessment of classifier's performance is also performed.

## 2. Crime Analysis

### 2.1. Data Collection

There is a collection of voluminous crime data in the police records. Every year, crime data from all over the nation are recorded in form of cases and the National Crime Bureau of Records keeps the availability of all such records. Usually, the collected data are unprocessed and have incorrect or missing values. To rectify these data and bring them in proper form, preprocessing of data is extremely significant. This involves the process of data cleansing and preprocessing.

### 2.2. Classification

The dataset is divided into several groups depending on some specific attributes of the data object. Based on the states and cities, the crime can be grouped. The process of classification involves classifying the crime depending on the different types of crime. Using the K-means algorithm, data having similar attributes can be grouped or clustered.

### 2.3. Pattern Identification

This process includes the identification of trends and patterns pertaining to the crime. The outcome of pattern identification is the crime pattern related to a specific place. As per the location, the relevant attributes are taken into accords such as weather conditions, significant event, area sensitivity, and existence of criminal groups. Such information related to the patterns supports the police officials to work smoothly and effectively.

### 2.4. Prediction

A model is built for the respective place. To fetch crime-prone areas, current date and attributes are fed into the prediction software. By the means of visualization, results are depicted.

### 2.5. Visualization

**Figure 1:**
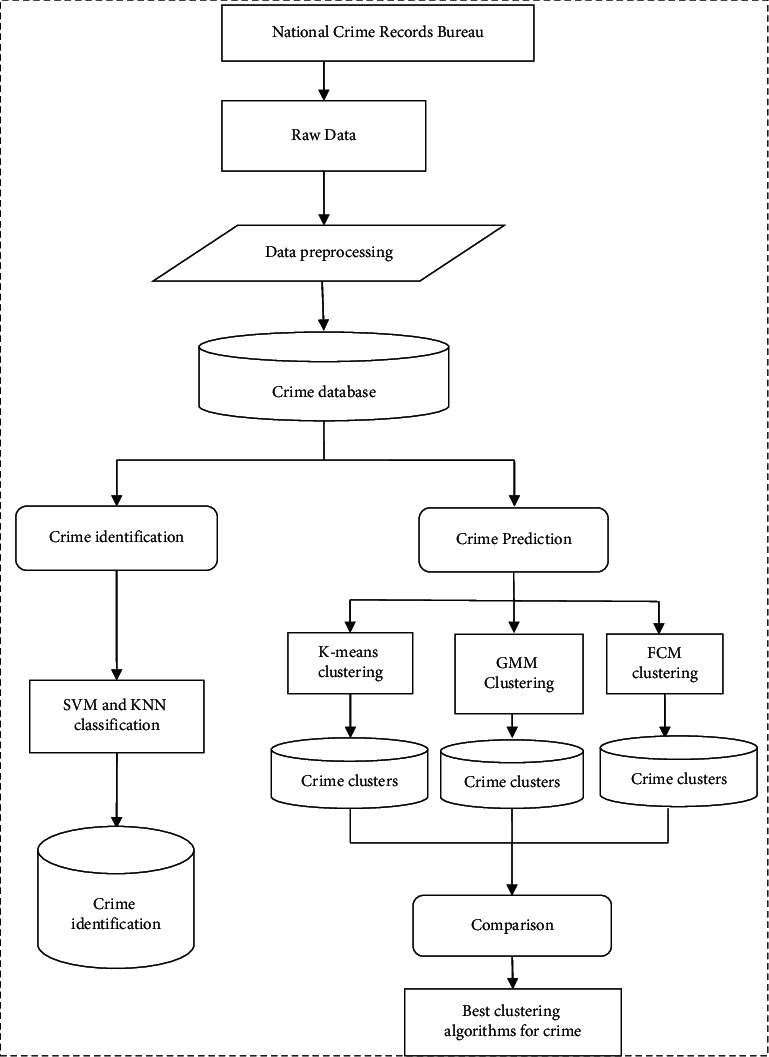
Crime analysis.

There is a graphical representation of the crime-prone areas through a heat map signifying the activity level. Dark colors depict low activity, whereas high activity is depicted using bright colors.  Figure 1 presents different phases of crime analysis.

Hackers tend to target underdeveloped nations much less frequently in general: on a continental level, Africa and Asia have the lowest rates of compromised email addresses (4 and 12 per 100 Internet users, respectively). The highest breach rates are in North America, where 1 in 2 Internet users experienced a breach in 2021. This figure exceeds the global average by three times. With two of every five Internet users penetrated, Oceania ranks second. The above data are obtained from https://surfshark.com/research/data-breach-impact/statistics.

## 3. Literature Survey

ML might be a subset of AI equipped for settling certifiable designing difficulties. It empowers gaining from information without the need of express programming. There is high coordination of ML in everyday life. Manjeet Rege et al. [1] suggested that ML strategies upheld the numerical models, information acquisitions, heuristic learning, and choice trees for performing choosing, in this way offering the advantages of solidness, controllability, and discernibleness. In the clinical field, refreshing is frequently done easily by attaching new patient's record.

Bharati et al. [2] recommended that, for finding human sickness, ML models assist the clinical experts with beginning phase indications. Marsland et al. [3] suggested that ML might be a division of AI that pushes forward the idea that, through giving get passage to legitimate information, machines can learn by utilizing themselves the best approach to tackle a particular issue. By receiving complex numerical and factual models, ML engages the machines to autonomously perform scholarly undertakings and settle on choices as opposed to depending on the conventional generally tackled by populace. Mechanization of complex undertakings by ML has profited extraordinarily inside the system administration field such as offloading of plan and activity of correspondence networks on the machines. There is effective execution of ML procedures in system administration regions such as interruption discovery proposed by Buczak et al. [4] and intellectual radios proposed by Bkassiny et al. [5].

In the midst of numerous system administration areas, the exploration stresses ML for optical system administration. Mukherjee et al. [6] recommended that for all the primary organization suppliers across the planet, optical organizations traverse the fundamental physical infra because of their various successful highlights such as lower cost, high limit, and undeniably more. DeCusatis [7] and Song et al. [8]proposed that it likewise entered the chief huge telecom showcases as datacom and thusly the entrance portion with no depressing odds of the other elective innovation as a swap for it inside not so distant future. Chatterjee et al. [9] and Talebiet al [10] proposed the complexity of various procedures, and approaches are assessed for upgrading the optical organization's presentation of optical. These incorporate the traffic prepping, directing, frequency task, and survivability of the basic transmission systems (from the perspective of system administration) projected during a succession of development in data plane and control plane. At the information plane, the methodology of EON has advanced as an inventive optical determination fit for reacting to the high versatility prerequisite in doling out optical organization assets. Dissimilar to the conventional fixed-framework WDM organizations, EON grants persistent and flexible data transfer capacity distribution. A speedy utilization of ML in optical systems administration is introduced inside the given exploration. Two of the exploration commitments incorporate, a basic instructional exercise on utilizing the ML techniques and their execution concerning the optical organizations and an overview of the momentum research work covering the subject additionally as leading an order of different used cases centered inside the investigation. Both optical correspondence and optical system administration are contemplated for energizing new cross-layer research headings. ML usage is broadly critical in cross-layer settings during a way that the information examination at the actual layer like checking BER induces alterations at the network layer like steering, range, and tweak design tasks. ML sending in optical correspondence and system administration is in a crude stage, and in this way, the survey covered inside the exploration stresses on allowing an initial reference for scientists and specialists enthusiastically getting acclimated with winning ML applications or investigating new examination draws near. The arising intelligent question inside the optical system administration space is that with a particularly exceptional development and application in ML for more than 30 years, there is a flood in its force now.

Supervised Learning: the preeminent well-known ML strategy is managed realizing, which takes into account the designing issues that were clarified by Xu and Yang [11]. It maps a gathering of info factors with a gathering of yield factors inside the most appropriate way. The framework understands to instill a capacity from a gathering of named preparing the informational collection, which includes a bunch of info highlights and different occurrence esteems for important credits. Jian-hua et al. [12] saw that upheld the managed learning, the foreseen execution exactness of ML calculation is frequently surveyed. The intention of the instilled work is to beat the trouble of relapse or arrangement. Different measurements utilized inside the estimation of the preparation task include explicitness, precision, affectability, kappa esteem, and region beneath the bend prior to beating any designing issue, and it is critical to choose a fitting calculation for finishing preparing relying on the information type. Since ML is information-driven, the strategy determination depends absolutely on such information. From that point comes the many periods of upgrading the picked ML calculations.

Skrinarova et al. [13] clarified that the classification task portrays an old-style issue concerning the information mining approach, which is at risk of dispensing a pre-indicated class to obscure information. Silva et al. [14] proposed a learning model that is established on the association in the midst of the indicator property estimations and accordingly the objective worth. The point lies in anticipating the class on the possibility of past scholarly information. ML alludes to such grouping issues as administered learning. Consequently, an information set should be made accessible including occasions with known classes and a test informational index that the classification should be chosen. Gao et al. [15] noticed that the characterization achievement exceptionally depends upon the information quality offered for learning close to the kind of ML calculation utilized. For instance, by fusing the grouping methods false clients applying for advance are regularly anticipated or the individuals who arrange mangoes almost as fortunate or unfortunate close by numerous other ongoing applications. The paired arrangement is the most noticeable kind of characterization issue during which the objective has two plausible estimations of yes or no, positive or negative, and so on. For estimating the exhibition of arrangement, different techniques are utilized such as the lift bend, disarray framework, and collector administrator attributes. Osisanwo et al. [16] analyzed that there is a particular learning strategy for every ML calculation relying on their boundaries esteems. While amending the order issue by utilizing a calculation and different arrangements of boundaries, there is a sudden distinction inside the grouping precision for each situation. The prime focal point of ML is to detect the worthy boundary estimations of the calculations, which helps in settling the designing issue concerning the exhibition measurements inside the best way. Consequently, it is necessary that the calculation boundaries are balanced and predictable with the issue to be settled. The PSO and search strategies are some of the improvement procedures. The examination accentuates on adjusting the calculation boundaries through format of investigation technique.

Veena and Meena [17] presented a method for analyzing a user's numerous identities and determining whether or not synthetic identity theft has occurred utilizing three types of data: input dataset (X), normal dataset (Y), and target dataset (Z).

The authors of the research such as Veena and Meena [18] employed four distinct methodologies to determine cybercrime. The detection of synthetic identity theft was first investigated. Secondly, the intrusion detection was checked using the honey pot security mechanism. Finally, the detection was improved by employing a lie detection algorithm that assessed a person's false speech. Finally, utilizing clustering algorithms, cybercrime was detected by analyzing the user profile. Veena et al. [19] proposed different techniques using the machine learning for the cybercrime detection.

The authors such as Veena and Meena [20] conducted a study on cyber warfare, which is currently the most serious threat to network security. The most difficult aspect of the network was dealing with security issues on the server side. To safeguard the network from intruders, the study proposed that security performance should be improved.

## 4. Research Methodology

### 4.1. Dataset

Cybercrime detection is based on a dataset obtained from the CBS open data StatLine. Open data are accessible for all tables in StatLine. A Web service (API) can be used to get the most recent version of a table, which can also be requested and downloaded in StatLine. The data can be handled automatically using the OData API. The personal data are secured in accordance with the European Union's General Data Protection Regulation (GDPR).

### 4.2. Supervised Learning Method

In a supervised learning method, a model is formed for predicting on the basis of evidence under uncertainty. With more number of observations, the predictive performance of the computer is also enhanced. A supervised learning algorithm considers the available group of input data and output in the form of known responses to the data. The overall input dataset resembles a heterogeneous matrix in which the rows are referred to as instances, observations, or examples, and the columns are referred to as attributes, predictors, or features. Both the row and column denote variables depicting a measurement done on each user.

`The response obtained is considered as the data in a column vector where each row comprises the output related to the respective observation in the input data. For training a supervised learning model, a suitable algorithm is employed, and thereafter, the input and response data are forwarded to it.

### 4.3. Support Vector Machine

This research section employs the cybercrime detection model through SVM for classifying the dataset retrieved from the CBS data StatLine https://www.cbs.nl/en-gb/our-services/open-data. Support vector machine helps in providing training. Once the classification is over, a particular user is predicted as Genuine or Crime User on the basis of several attributes.

Steps are as follows:

 
*Step 1*. Real-time dataset is input.
 
*Step 2*. Classification is performed using the clustering techniques.
 
*Step 3.* Classification is carried out through SVM.
 
*Step 4.* Based on the average acquired from the data, cluster classification is performed. Also, based on new classes SVM classifier is conducted for different attributes /predictors /features that is 10.
 
*Step 5*. For carrying out performance evaluation, various performance metrics are employed such as TP, FP, TN and FN, FAR, ACC—accuracy, DR—detection rate, specificity, sensitivity, precision, recall, and Fowlkes–Mallows scores for different attributes.
 
*Step 6*. Thereafter, the following are determined for the training data: cvMSE—mean-squared error for regression via 10-fold cross-validation, cv MCR—misclassification rate via stratified 10-fold cross-validation, and cfMat—confusion matrix via stratified 10-fold cross-validation. SVM Struct. Support Vectors, SVM Struct. Alpha, SVM Struct. Bias, and SVM Struct. Support Vectorization are obtained for the training data along with finding min and max values for the training attributes.
 
*Step 7.* With the use of SVM, the classification accuracy of 89% is achieved.

### 4.4. SVM Classifier Training Data

SVM classifier makes use of cybercrime detection datasets through ML tools.  Table 1 depicts training data for SVM classifier.

### 4.5. KNN Classifier

The KNN technique is utilized to conduct classification and regression in this case. The KNN approach is used to estimate continuous variables in KNN regression. A weighted average of the *k* closest neighbors is another method. In this work, the value of *k* is 2. The labelled examples are arranged in order of increasing distance neighbors by the inverse of their distance. The algorithm's operation is as follows: determining the Euclidean distance between the query and labelled examples.

### 4.6. Comparison of SVM and KNN Classifier

In KNN, data categorization is based on the distance metric, while in SVM, the correct training phase is necessary. Because SVM is of the ideal kind, it ensures that the divided data are segregated optimally. KNN is typically used as a multiclass classifier, whereas SVM is used to separate binary data into one of two classes. For a multiclass SVM, the one-vs-one and one-vs-all approaches are used. *n*∗(*n* − 1)/2 SVMs are trained on the one-vs-one idea, which means one SVM for each pair of classes. The entity is fed a pattern that is unknown to it, and the data type is determined by the majority output from the aggregate SVM output. This method is primarily used in multiclass categorization. The data are classified as Genuine data or Crime data. The Genuine data are the users 1–32, 49, 51, 53–55, 57–96, and 98–100. The rest are the crime data.

SVMs appear to be computationally demanding, as the model may be used to predict classes even when additional unlabeled data are encountered once the data have been trained. In the case of KNN, however, the distance metric is computed every time new unlabeled data are encountered. In KNN, just the K parameter must be fixed, and the distance metric must be appropriate for classification, however in SVMs, the *R* parameter.

Regularization term must be chosen together with the kernel parameters if the classes are linearly inseparable. In contrast to KNN, SVMs show improved accuracy when comparing the accuracy of both classifiers (Table 2).

## 5. Unsupervised Learning Method

Unsupervised learning is a self-controlled Hebbian learning capable of identifying past unknown patterns in dataset without preexisting labels. This approach is also referred to as self-organization that permits forming probable densities of given inputs. Unsupervised learning is an integral part of ML along with two other techniques of supervised and reinforcement learning. Principal component and cluster analysis are the two main methods adopted in unsupervised learning.

### 5.1. Clustering Techniques

Unsupervised learning is a form of ML algorithm that derives inferences from datasets comprising input data and no labelled responses. Cluster analysis is a popular unsupervised learning method employed for exploratory data analysis for identifying hidden patterns or clustering of data.

### 5.2. Clustering with the Gaussian Mixtures Model Using EM Algorithm

#### 5.2.1. EM for Factor Analysis

(1) $$\begin{array}{c} \text{The probable log possibility for factor analysis is }Q=E\left[\text{log}\prod_{i}{\left(2\pi \right)}^{p/2}{\left|\psi \right|}^{-\left(1/2\right)}\text{expexp }\left\{-\frac{1}{2}\left[x_{i}-A_{z}\right]\psi^{-1}\left[x_{i}-A_{z}\right]\right\}\;\right], \end{array}$$

(2) $$\begin{array}{c} =c-\frac{n}{2}\text{log}\left|\psi \right|-\sum_{i}E\left[\frac{1}{2}x_{i}^{\prime }{\left|\psi \right|}^{-1}x_{i}-x_{i}^{\prime }\psi^{-1}Az+\frac{1}{2}Z^{\prime }A^{\prime }\psi^{-1}Az\right] \end{array}$$

Here, *c* denotes a constant, which is not dependent on the parameters, and tr denotes the trace operator.

### 5.3. Finite Mixture Models

In the available dataset *D* = {*x*_1_ ,. . ., *x*_*n*_}, *xi* denotes a d-dimensional vector measurement. The points are presumed to be generated in an IID manner from an underlying density *p*(*x*). An assumption is made that *p*(*x*) denotes a finite mixture model having *K* components, in which

(3) $$\begin{array}{c} P\left(\underline{x}|\theta =\sum_{K=1}^{K}a_{k}p_{k}\left(\underline{x}|z_{k},\theta_{k}\right)\right), \end{array}$$

where

- $p_{k}\left(\underline{x}|z_{k}\;,\theta_{k}\right)\;$ depicts mixture components, and 1 ≤ *k* ≤ *K* represents density or distribution defined over *p*(*x*), with parameters *θ*_*k*_.
- *z* = (*z*_1_,. . ., *z*_*k*_) represents a vector of *K* binary indicator variables, which are mutually exclusive and exhaustive. *z* is a *K*-ary random variable, which depicts the identity of the mixture component that generated *x*. It is easier for mixture models for depicting *z* as a vector of *K* indicator variables.
- *a*_*k*_  *=* *p(z*_*k*_) depicts the mixture weights and the probability that an arbitrarily chosen *x* was produced by *k* component, where ∑_*k*=1_^*k*^*a*_*k*_=1.
- The overall parameter set for a mixture model including K components is Θ = {*a*_1_*, . . .,a*_*k*_,*θ*_*k*_*, . . .,θ*_*k*_}.

### 5.4. GMM

For $\underline{x}$*∈* Rd, a Gaussian mixture model is defined by formulating every K component a Gaussian density with parameters ${\underline{µ}}_{k}$ and Σ_*k*_. Each component represents a multivariate Gaussian density.

#### 5.4.1. The EM Algorithm for GMM

Expectation-maximization (EM) algorithm for Gaussian mixtures is defined as follows. The algorithm resembles an iterative algorithm initializing from some beginning estimate of Θ (e.g., random), and thereafter, proceeding to repetitively update Θ until the identification of convergence, every iteration comprises an E-step and an M-step.

*E-Step*. It represents the existing parameter values as Θ. *w*_*ik*_ is computed concerning all data points, 1 ≤ *i* ≤ *N,* and all mixture components 1 ≤ *k* ≤ *K*. For every data point (xi), the defined membership weights are depicted as ∑_*K*=1_^*K*^*w*_*ik*_  = 1. This results in an *N* × *K* matrix of membership weights, with every row summing up to 1.

*M-Step*. The membership weights and the data are utilized for computing new parameter values. Let *N*_*k*_  *=*  ∑_*i*=1_^*N*^*w*_*ik*_, which represents the sum of the membership weights for the kth component. This signifies the valid number of data points allocated to component *k*.

(4) $$\begin{array}{c} a_{k}^{\text{new}}=\frac{N_{k}}{N},\;1\leq k\leq k. \end{array}$$

### 5.5. Reasons for Choosing the Gaussian Clustering Technique

GMMs presume a specific amount of Gaussian distributions wherein each distribution depicts a cluster. Therefore, a Gaussian mixture model works by grouping the data points pertaining to a single distribution.

For instance, the scatter plot displaying two clusters in blue color and certain fringe observations in red color that being part of any of the two clusters is considered. By being extremely genuine to the data, observations are allocated to the clusters. The data can be defined more precisely by permitting partial assignment to various clusters.

**Figure 2:**
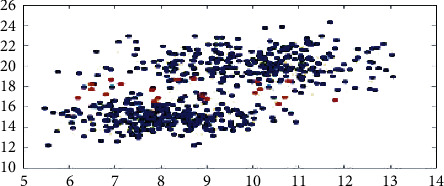
Gaussian mixture models.

This can be performed by soft clustering of the data. Soft clustering is also referred to as fuzzy clustering in which observations are a part of multiple clusters. The present research discusses a soft clustering technique referred to as EM of a Gaussian mixture model (Figure 2).

### 5.6. Gaussian Function

The Gaussian function-based distance measure has been considered for determining the similarity amidst the data samples of the intrusion dataset. The same distance measure is utilized, and the data samples are clustered using the K-means algorithm. To minimize dimensionality, K-means clustering is adopted for acquiring clusters through the recommended distance function.

Thereafter, the distance amidst each training data sample and each cluster of centroids is computed. Subsequently, the nearest neighbor is determined for every sample data sample in the cluster. By summing up these two distances, a new distance value is obtained. For every training data sample, the distance value depicts a single feature. Hence, there is a mapping of each data sample of the training set to a single feature value, thus minimizing the dimensionality to 1. The following equation defines the suggested distance function.

The function *G*(*x*, *µ*, *σ*) is defined as follows:

(5) $$\begin{array}{c} G\left(x,\;µ\;,\sigma \right)=\left\{e^{-\left(\left(x-u\right)/\sigma \right);\text{ one or both system calls exist}}\;0{\;}^{;\text{none of the system calls exist}}\right., \end{array}$$

where *x* = system call taken in regard.*µ* = mean of the system call related to data samples available in the cluster.*σ* = standard deviation of the system call related to the data samples of the training set.

### 5.7. K-Means Clustering

The K-means algorithm has drastically progressed and adopted because of its mode of operation. The algorithm works by clustering the observations into *k* groups, wherein *k* acts as the input parameter. Thereafter, each observation is allocated to the clusters on the basis of the observation's proximity to the cluster's mean. The mean value is further calculated again, and the process restarts. Following is the working of Algorithm 1.

### 5.8. Cluster Quasi-Random Data Using FCM

FCM signifies a data clustering technique in which every single data point is a part of a cluster to a certain extent as indicated by a membership grade. Jim Bezdek in 1981 originally proposed this technique, which is a reformed version of the previous clustering methods. The method involved in this technique helps in grouping data points that populate a part of the multidimensional space into a certain number of different clusters. For every data point, the command line function denoted as “fcm” results in various cluster centers and membership grades for the respective data point. This information is returned by the fcm function for enabling fuzzy inference system to build membership functions for depicting the fuzzy qualities of each cluster.

## 6. Performance Evaluation of the Proposed Research Using Real-Time Dataset

Many of the researchers keep a repository of various types of data from their studies and share it with community repositories. The current part uses machine learning and artificial intelligence research to characterize the most common security-related datasets.

### 6.1. Collection of Cybercrime Data

A variety of cybercrime data is obtained by evaluating the crime pattern to predict cybercrime in the banking industry. The information comes from a variety of online sources, including news feeds, blogs, articles, and police department websites. The acquired cybercrime data are subsequently saved in a crime database for further processing.

### 6.2. Preprocessing of Cybercrime Dataset

The cybercrime dataset stored that is stored in the crime database must undergo preprocessing before the data mining techniques are applied to it. By performing preprocessing, missing values, noisy data, etc., can be worked upon.

### 6.3. Data Mining Techniques

Data mining techniques and algorithms are applied to preprocessed data to detect any fraud using knowledge innovation from sudden patterns, hence combating cyber credit card fraud. Data mining is a useful method for addressing challenges in the banking industry by uncovering hidden patterns, linkages, and relationships in corporate data acquired from crime databases.

## 7. Evaluation of Performance of Classifier

For evaluating the efficiency of IDS, various kinds of metrics that have been formulated are classified into three classes and these are threshold, ranking, and probability metrics. Threshold metrics comprises CR, F-measure, CPE (cost per example), etc. The forecast is either above or below the threshold, and it does not have to be near to one; the threshold metric value ranges from 0 to 1. FPR, PR—precision, DR—detection rate, CID—intrusion detection capability, and AUC—area under ROC curve are ranking measures with values ranging from 0 to 1. These metrics are based on how the examples are ordered rather than the actual anticipated values. It makes no impact till the ordering is maintained. These metrics evaluate the proper ordering of the attack instances prior to the normal instances and are observed as an outline of model performance pertaining to overall thresholds. The root-mean-square error (RMSE) is a probability statistic with values ranging from 0 to 1. When the anticipated value of each attack class equals the genuine conditional likelihood of that class being a normal class, the metric declines. There is a comparison of different IDSs with well-known metrics such as AUC. The CID value is a number that varies from 0 to 1. The CID value has a direct relationship with IDS performance. That is, a high CID value equates to a high IDS rating. The confusion matrix is frequently used to aid in the computation of these measures. The confusion matrix is quite helpful in representing the IDS classification output.

### 7.1. Metrics from Confusion Matrix

Though the confusion matrix is highly helpful in representing the classification, it is not adequate and significant enough for comparing the IDSs. To combat this issue, several performance metrics are described with respect to the confusion matrix variables.

There may be two sources for the delay and chaff perturbations produced in the attack flow: first being the attacker and second being the network itself.

### 7.2. Evaluation of Performance of SVM Classifier

The performance analysis of the SVM classifier is performed by making use of the following metrics:

(6) $$\begin{array}{c} \text{FAR}=\frac{\text{FP}}{\text{FP}+\text{TN}},\\ \text{ACC}=\frac{\text{TP}+\text{TN}}{\text{TP}+\text{TN}+\text{FP}+\text{FN}},\\ \text{RECALL}=\frac{\text{FP}+\text{FN}}{\text{Total User}},\\ \text{Specificity}=\frac{\text{TP}}{\text{TP}+\text{FN}},\\ \text{Sensitivity}=\frac{\text{TN}}{\text{FP}+\text{TN}}. \end{array}$$

*Fowlkes–Mallows Scores*. The Fowlkes–Mallows index is utilized on knowing the ground truth class assignments of the samples. The Fowlkes–Mallows score FMI is stated as the geometric mean of the pairwise precision and recall:

(7) $$\begin{array}{c} \text{FMI}=\frac{\text{TP}}{\sqrt{\left(\text{TP}+\text{FP}\right)\left(\text{TP}+\text{FN}\right)}}. \end{array}$$

Table 3 depicts the performance metrics of SVM classifier, and Table 4 depicts performance metrics pertaining to cross-validation partition.

## 8. Conclusion

This research work elaborates crime analysis, supervised learning methods incorporating SVM and KNN classifier and their comparison, unsupervised learning methods such as cluster with Gaussian mixture model making use of EM algorithm, motive behind selecting K-means clustering, Gaussian clustering techniques, and cluster quasi-random data through FCM. In addition, it evaluated the user profile by the means of several clustering techniques for the identification of cybercriminal.

Machine learning algorithms exhibiting superior performance are evaluated using multiple datasets. According to the research work investigation, the performance of Gaussian clustering technique surpasses the remaining clustering techniques in the unsupervised mode. It is evident from the results that the detection of cybercrime can be done precisely.

Eventually using the best technique, the criminal is identified and the Gaussian mixture model in the unsupervised method reveals enhanced performance using the detection method. 96.56% percentage of accuracy is achieved in detecting the criminal. The accuracy achieved in case of classification via SVM classifier is 89% in the supervised method.

The ultimate goal was to enhance the security performance of the network so that it can be safeguarded from the attackers. With the help of cluster computing techniques and real-time dataset, the performance evaluation of several cybercriminal detection methods is analyzed. The assessment of classifier's performance is also performed. This work is done using the dataset. Hence, it can be concluded that it is feasible to recognize cybercrime that has been artificially created using machine learning or another method. Also, it is better to be alert and prevent being victim of cybercrime.

## Figures and Tables

**Algorithm 1:**
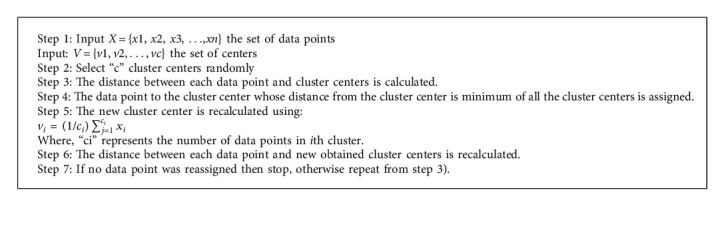
K-Means algorithm.

**Table 1:** Training data for the SVM classifier.

Sl. no.	Training dataset (average)	SVM Struct. Support Vectors	SVM Struct. Scale Data Shift	SVM Struct. Scale Data Scale Factor	SVM Struct. Alpha	SVM Struct. Bias	SVM Struct. Support Vectorization	Mean-squared error for regression using 10-fold cross-validation:	Misclassification rate using stratified 10-fold cross-validation:	Confusion matrix using stratified 10-fold cross-validation:
								cvMSE	cvMCR	cfMat
1	7.850	0.314	−7.102	0.260	0.625	1.958	6.000	0.003	0.150	0 1 0 12 69 2 0 0 16
2	8.040	0.403	−7.102	0.260	0.625	1.958	10.000	0.003	0.150	
3	8.000	0.379	−7.102	0.260	0.625	1.958	11.000	0.003	0.150	
4	7.750	0.317	−7.102	0.260	0.625	1.958	13.000	0.003	0.150	
5	7.730	0.462	−7.102	0.260	0.625	1.958	14.000	0.003	0.150	
6	8.310	0.384	−7.102	0.260	0.625	1.958	15.000	0.003	0.150	
7	7.780	0.743	−7.102	0.260	0.625	1.958	49.000	0.003	0.150	
8	7.670	0.936	−7.102	0.260	−1.250	1.958	50.000	0.003	0.150	
9	8.220	0.749	−7.102	0.260	0.625	1.958	51.000	0.003	0.150	
10	8.650	0.837	−7.102	0.260	−2.500	1.958	52.000	0.003	0.150	
11	8.560	0.533	−7.102	0.260	0.625	1.958	53.000	0.003	0.150	
12	8.170	0.689	−7.102	0.260	0.625	1.958	54.000	0.003	0.150	
13	8.320	0.754	−7.102	0.260	0.625	1.958	55.000	0.003	0.150	
14	8.880	0.790	−7.102	0.260	−2.500	1.958	56.000	0.003	0.150	
15	8.580	0.319	−7.102	0.260	0.625	1.958	62.000	0.003	0.150	
16	8.180	0.907	−7.102	0.260	−2.500	1.958	97.000	0.003	0.150	
17	7.320	0.561	−7.102	0.260	0.625	1.958	98.000	0.003	0.150	
18	7.230	0.343	−7.102	0.260	0.625	1.958	100.00	0.003	0.150	

**Table 2:** TP, TN, FN, and FP classification for attribute.

UID	Group		Attribute 1					
	Cluster classification based on average	GD = 0	New class SVM classifier: Attribute 1	GD = 0	TP	TN	FP	FN
		CD = 1		CD = 1	0	1	11	10
1	Genuine User	0	“Crime User”	1	FALSE	TRUE	FALSE	FALSE
2	Genuine User	0	“Crime User”	1	FALSE	TRUE	FALSE	FALSE
3	Genuine User	0	“Crime User”	1	FALSE	TRUE	FALSE	FALSE
4	Genuine User	0	“Crime User”	1	FALSE	TRUE	FALSE	FALSE
5	Genuine User	0	“Crime User”	1	FALSE	TRUE	FALSE	FALSE
6	Genuine User	0	“Crime User”	1	FALSE	TRUE	FALSE	FALSE
7	Genuine User	0	“Crime User”	1	FALSE	TRUE	FALSE	FALSE
8	Genuine User	0	“Crime User”	1	FALSE	TRUE	FALSE	FALSE
9	Genuine User	0	“Crime User”	1	FALSE	TRUE	FALSE	FALSE
10	Genuine User	0	“Crime User”	1	FALSE	TRUE	FALSE	FALSE
11	Genuine User	0	“Crime User”	1	FALSE	TRUE	FALSE	FALSE
12	Genuine User	0	“Crime User”	1	FALSE	TRUE	FALSE	FALSE
13	Genuine User	0	“Crime User”	1	FALSE	TRUE	FALSE	FALSE
14	Genuine User	0	“Crime User”	1	FALSE	TRUE	FALSE	FALSE
15	Genuine User	0	“Crime User”	1	FALSE	TRUE	FALSE	FALSE
16	Genuine User	0	“Crime User”	1	FALSE	TRUE	FALSE	FALSE
17	Genuine User	0	“Crime User”	1	FALSE	TRUE	FALSE	FALSE
18	Genuine User	0	“Crime User”	1	FALSE	TRUE	FALSE	FALSE
19	Genuine User	0	“Crime User”	1	FALSE	TRUE	FALSE	FALSE
20	Genuine User	0	“Crime User”	1	FALSE	TRUE	FALSE	FALSE
21	Genuine User	0	“Crime User”	1	FALSE	TRUE	FALSE	FALSE
22	Genuine User	0	“Crime User”	1	FALSE	TRUE	FALSE	FALSE
23	Genuine User	0	“Crime User”	1	FALSE	TRUE	FALSE	FALSE
24	Genuine User	0	“Crime User”	1	FALSE	TRUE	FALSE	FALSE
25	Genuine User	0	“Genuine User”	0	TRUE	FALSE	FALSE	FALSE

**Table 3:** Performance metrics using the SVM classifier.

Attribute	TP	TN	FP	FN	FAR	DR	ACC	Precision	Recall	Specificity	Sensitivity	FMI
1	16	64	20	0	0.2381	1	0.8	0.23810	0.2	1	0.7619	0.6667
2	16	64	20	0	0.2381	1	0.8	0.23810	0.2	1	0.7619	0.6667
3	80	0	9	11	1.00	0.879	0.8	2.22222	0.2	0.8791	0.00	0.8889
4	80	0	16	4	1.00	0.952	0.8	1.25000	0.2	0.9523	0.00	0.8909
5	80	0	0	20	0.00	0.8	0.8	0.00000	0.2	0.8	0.00	0.8944
6	80	0	0	20	0.00	0.8	0.8	0.00000	0.2	0.8	0.00	0.8944
7	80	0	0	20	0.00	0.8	0.8	0.00000	0.2	0.8	0.00	0.8944
8	80	0	0	20	0.00	0.8	0.8	0.00000	0.2	0.8	0.00	0.8944
9	33	47	20	0	0.298	1	0.8	0.29851	0.2	1	0.7014	0.7891
10	80	0	0	20	0.00	0.8	0.8	0.00000	0.2	0.8	0.0000	0.8944

**Table 4:** Performance metrics using the SVM classifier in cross-validation partition.

Cross-validation partition										
Attributes used	cvMSE	cvMCR	cfMat			Type	*N*	Num test sets	Train size	Test size
1,2,3,8	0.0025	0.15	0	1	0	K-fold	100	10	90	10
1,2,3,4	0.0025	0.03	12	69	2					
			0	0	16					

## Data Availability

For carrying out cybercrime detection, the dataset is fetched from CBS open data StatLine (https://www.cbs.nl/en-gb/ourservices/open-data and https://www.cbs.nl/en-gb/our-services/customisedservices-microdata/microdata-conducting-your-own-research) and CBS open datasets (https://www.dataworld.com/en/home/ and https://www.kaggle.com/datasets).

## Abbreviations

CSVM: Classifier support vector machine
KNN: K-nearest neighbor
TP: True positive
FP: False positive
TN: True negative
FN: False negative
FAR: False alarm rate
DR: Detection rate
ACC: Accuracy
EM: Expectation maximization
cv: Cross-validation
MSE: Mean-squared error
MCR: Misclassification rate
GD: Genuine data
CD: Crime data
UIDv: User ID
PCA: Principal component analysis
ROC: Receiver operator characteristics
GMM: Gaussian mixture model
k: Input parameter *S* = *s*1, s2,…. - preclassified samples Si = *x*1, x2... – vector
cfMat: Confusion matrix
EON: Elastic optical network
WDM: Wavelength division multiplexing
BER: Bit error rate
PSO: Particle swarm optimization.
